# Clinical Pharmacogenetics: Results After Implementation of Preemptive Tests in Daily Routine

**DOI:** 10.3390/jpm15060245

**Published:** 2025-06-10

**Authors:** Xando Díaz-Villamarín, María Martínez-Pérez, María Teresa Nieto-Sánchez, Emilio Fernández-Varón, Alicia Torres-García, Isabel Blancas, José Cabeza-Barrera, Rocío Morón

**Affiliations:** 1 Instituto de Investigación Biosanitaria de Granada (Ibs. Granada), 18012 Granda, Spain; 2 Hospital Pharmacy, Hospital Universitario San Cecilio, 18016 Granada, Spain; 3 Department of Pharmacology, Center for Biomedical Research (CIBM), University of Granada, 18016 Granada, Spain; 4 Medical Oncology, Hospital Universitario San Cecilio, 18016 Granada, Spain

**Keywords:** pharmacogenetics, pharmacogenomics, personalized medicine, clinical implementation, hospital pharmacy, clinical pharmacy, preemptive pharmacogenetics

## Abstract

**Background/Objectives:** The clinical implementation of pharmacogenetics (PGx) remains limited, even for well-established drug–gene interactions. In addition to insufficient infrastructure and PGx education among healthcare professionals, there is currently no consensus regarding which genetic variants should be tested, the most appropriate testing approach (e.g., single-gene vs. multi-gene panels), or how to translate genotypes into actionable therapeutic recommendations. **Methods:** We describe the implementation of PGx in real daily clinical routine at a single institution to guide other centers. We analyze the drug–gene interactions and genetic variants included in our program based on allelic, genotypic, and phenotypic frequencies, resulting therapeutic recommendations. Linkage disequilibrium and haplotype analyses are also performed. **Results and Conclusions:** PGx testing was primarily requested by the oncology department. Not all variants included in typical panels had clinical utility in our setting. We do not recommend testing CYP2C19*17 prior to clopidogrel prescription, as it does not translate into a dosing recommendation. TPMT*3B may be considered just to confirm TPMT*3A due to its linkage with TPMT*3C. Similarly, we do not recommend the routine testing of CYP2C9*2 prior to siponimod prescription, as it does not inform therapeutic decisions according to the current drug label.

## 1. Introduction

Pharmacogenetics (PGx) enables the prediction of interindividual variability in drug response, thereby supporting the individualization of treatments and bringing personalized medicine closer to routine clinical practice. Understanding how genetic variants—particularly single-nucleotide polymorphisms (SNPs)—influence drug efficacy or toxicity allows physicians to tailor pharmacological treatments when multiple therapeutic options are available.

Although the clinical implementation of pharmacogenetics has been widely discussed in recent years, several barriers still hinder its widespread adoption [1]. The main constraint is the lack of training and education among healthcare professionals in genetics, particularly in PGx. Other significant barriers include the absence of adequate technical infrastructure, uncertainty about which genetic variants should be tested for each patient, the complexity of translating genotypes into phenotypes and clinical or dosing recommendations, the heterogeneity among existing dosing guidelines based on PGx data, and bioethical concerns related to the handling of genetic material and data protection.

On the other hand, it is known that nearly 15% of medicinal products approved by the European Medicines Agency (EMA) or the U.S. Food and Drug Administration (FDA) up to 2014 include PGx information in their drug labeling [2]. Both agencies have emphasized that the genotyping of multiple genetic variants should be considered prior to initiating treatment with certain drugs. Notably, the FDA recently published a list of more than 50 medicinal products with PGx associations for which available data support therapeutic management recommendations [3]. In addition, PGx-based dosing guidelines are available through organizations such as the Clinical Pharmacogenetics Implementation Consortium (CPIC) [4] and the Dutch Pharmacogenetics Working Group (DPWG) [5], established by the Royal Dutch Pharmacists Association. Both sets of guidelines can be easily accessed via the Pharmacogenomics Knowledge Base (PharmGKB) [6]. Moreover, the cost of genotyping has decreased significantly in recent years, multiple studies have demonstrated the cost-effectiveness of various PGx tests [7,8], and several private companies have incorporated such tests into their service offerings.

To help overcome these barriers, a brochure detailing the PGx tests included in the Common Portfolio of Services of the Spanish National Health System was published in 2023. Its objective is to ensure more homogeneous and equitable access to genetic testing across the country by identifying the drug–gene interactions that should be offered for testing in hospitals through the public healthcare system. Notably, this portfolio represents the first nationally approved PGx catalog within a European healthcare system, marking a significant milestone in the integration of genetics into routine clinical practice.

### 1.1. Strategies and Methodology for PGx Implementation in Clinical Routine

Depending on the stakeholders involved in the healthcare process, various strategies for implementing PGx and personalized medicine have been proposed and attempted. Preemptive PGx testing strategies have been led by hospital and community pharmacies, primary care physicians, and medical specialists, depending on the clinical context and organizational model.

In addition, various industry stakeholders have developed PGx panels that include all relevant genetic variants supported by FDA and/or EMA data. Others have created more focused panels, targeting variants relevant to specific hospital departments—for example, cardiovascular PGx panels. In many clinical scenarios, testing is limited to a single drug–gene interaction prior to prescription.

Using multi-gene panels provides broader information that may benefit patients in the future by streamlining treatment decisions and avoiding the need for additional testing. However, this approach is more time-consuming, costly, and typically requires larger sample volumes. In contrast, testing a single drug–gene interaction is faster, more affordable, and can often be performed using minimally invasive samples such as saliva. Nevertheless, this strategy may require additional PGx testing if new pharmacological treatments are initiated later on. These trade-offs have been discussed in recent systematic reviews evaluating the cost-effectiveness of different PGx testing strategies in clinical practice [9,10].

Regardless of the chosen strategy, clinical decision support systems (CDSSs) are available to manage and integrate PGx data into clinical workflows [11,12]. These systems provide all the necessary information to optimize pharmacotherapy, even if new medications are prescribed to the patient in the future.

Moreover, PGx implementation can also differ depending on the clinical motivation behind the test request. Although PGx testing is highly valuable as a preemptive tool to improve treatment efficacy and reduce adverse drug reactions, many physicians still request it retrospectively, primarily as an explanatory tool after an adverse drug event (ADE) has occurred.

Despite the diversity in implementation strategies, there is substantial evidence supporting the clinical utility and applicability of PGx knowledge in daily practice.

### 1.2. Relevant Drug–Gene Interactions

A large number of genetic variants have been associated with variable drug responses, many of them supported by the highest levels of clinical evidence. In some cases, the drug label includes a description of the drug–gene interaction, although only a subset provides therapeutic recommendations based on the patient’s genotype.

Clopidogrel is an antiplatelet prodrug whose activation partially depends on the hepatic enzyme CYP2C19. Genetic variants in the gene encoding this enzyme have been associated with an altered drug response. Specifically, CYP2C19*2 (rs4244285) and CYP2C19*3 (rs4986893) are loss-of-function (LOF) alleles that result in reduced formation of the active clopidogrel metabolite, leading to decreased platelet inhibition and an increased risk of cardiovascular events [13,14]. CPIC guidelines recommend considering alternative antiplatelet therapy in intermediate metabolizers (IMs), who carry one LOF allele, and poor metabolizers (PMs), who carry two LOF alleles, due to their impaired response to clopidogrel [15].

Conversely, the CYP2C19*17 allele (−806C > T; rs12248560) is associated with increased enzyme activity, known as a gain-of-function (GOF) variant [16,17]. Individuals with two CYP2C19*17 alleles are classified as ultra-rapid metabolizers (UMs). However, PGx dosing guidelines for clopidogrel do not currently provide specific recommendations for these patients [18]. Despite this, the CYP2C19*17 allele may be clinically relevant in other therapeutic contexts, such as treatment with voriconazole. For UMs (CYP2C19*17/*17), CPIC guidelines recommend selecting an alternative antifungal not metabolized by CYP2C19, as achieving therapeutic drug concentrations may be difficult. In contrast, for PMs, the risk of adverse events increases, necessitating dose adjustment or alternative therapy [19]. The DPWG guidelines offer a different approach, recommending a 50% increase in the standard voriconazole dose with therapeutic drug monitoring during the first five days to guide dose adjustment [20]. These divergent recommendations highlight the complexity of PGx-guided therapy and underscore the need to tailor decisions based on both genotype and drug characteristics.

The TPMT and NUDT15 enzymes are responsible for the metabolism of azathioprine. Several SNPs in the *TPMT* and *NUDT15* genes result in reduced enzymatic activity, leading to the accumulation of active metabolites and an increased risk of myelosuppression. Carriers of at least one TPMT*2 (rs1800462) allele, two TPMT*3B (rs1800460) or TPMT*3C (rs1142345) alleles, or one TPMT*3A allele (combining rs1800460 and rs1142345) are at a significantly higher risk of severe toxicity when treated with standard thiopurine doses [21].

In oncology, numerous PGx associations have been identified. Variants in *DPYD*, *UGT1A1*, and *CYP2D6* have been linked to variable responses to capecitabine/5-fluorouracil (5-FU) [22], irinotecan [23], and tamoxifen [24], respectively. For these drug–gene interactions, well-established PGx-based dosing guidelines are available, and several regulatory authorities, including the EMA, have recommended genetic testing prior to treatment initiation [25].

Importantly, drug labels for clopidogrel, azathioprine, capecitabine/5-FU, irinotecan, and tamoxifen all include recommendations to genotype the relevant enzymes (*CYP2C19*, *TPMT*, *DPYD*, *UGT1A1*, and *CYP2D6*, respectively).

Furthermore, some drug labels include mandatory PGx testing. For example, siponimod—the first oral treatment approved for adults with secondary progressive multiple sclerosis—is contraindicated in patients with a CYP2C9*3/*3 genotype. In patients with CYP2C9*1/*3 or *2/*3 genotypes, a reduced maintenance dose of 1 mg daily is recommended.

### 1.3. Relevance of Allelic, Genotypic, and Phenotypic Frequencies in PGx Implementation in Clinical Practice

Many of the most clinically relevant PGx genes contain a large number of genetic variants, many of which are individually rare. However, due to the presence of multiple variants within the same gene, the overall likelihood of carrying at least one variant that impacts drug response and requires a dosing adjustment increases significantly. It is estimated that approximately 95% of patients carry at least one pharmacogenetically relevant polymorphism or mutation [26], and this percentage continues to grow as new variants are identified.

The allelic frequencies of PGx-relevant polymorphisms often vary widely across populations. In fact, an allele considered minor in one population may be the major allele in another [26, 27] Many DNA variants have a minor allele frequency (MAF) ranging from >1% to <0.001%, depending on ethnic background. As more variants are discovered and linked to drug response, the proportion of patients with potentially suboptimal pharmacological outcomes increases exponentially.

In addition, linkage disequilibrium (LD) between PGx-relevant SNPs can affect the interpretation of genetic tests. If two variants are always inherited together and the resulting phenotype remains unchanged regardless of whether one or both are present, testing both may be redundant. Moreover, some PGx variants deviate from the Hardy–Weinberg equilibrium (HWE) when tested in PGx studies, particularly when the variants are associated with the underlying disease rather than with drug metabolism.

Therefore, understanding the distribution of genotypes, alleles, and associated phenotypes of “ready-to-implement” PGx variants under real-world clinical conditions is essential. This knowledge can facilitate the effective integration of PGx into clinical practice and help to assess its practical utility.

## 2. Materials and Methods

### 2.1. Aim of the Study

The aim of this study is to describe the implementation of PGx in real-world clinical practice, to provide a practical guide for PGx implementation, and to evaluate the clinical relevance of the drug–gene interactions and genetic variants tested in our hospital. This evaluation is based on the observed allelic, genotypic, and phenotypic frequencies, as well as the associated dosing recommendations, in a population of patients treated with at least one medication guided by an available PGx test.

### 2.2. Design

We conducted a retrospective descriptive analysis of the PGx tests performed in our hospital since the establishment of the PGx unit. The analysis included genotypes, phenotypes, and the resulting therapeutic recommendations. The study population comprised patients from the Iberian Peninsula who had been prescribed a drug for which a PGx test was available at our center.

To assess the relevance of the tested variants for PGx implementation, we compared the genotypic and allelic frequencies in our population with those reported by the 1000 Genomes Project [27]. No formal sample size calculation was performed, as this study was designed as a retrospective descriptive analysis aimed at characterizing the implementation process and clinical applicability of PGx testing in real-world routine practice. No inferential comparisons or hypothesis testing were conducted. In addition, we performed a linkage disequilibrium (LD) analysis of PGx biomarkers for each gene included in the panel, provided that at least two different polymorphisms were tested and there was at least one patient carrying the variant. A haplotype analysis was also carried out to identify the most frequent SNP combinations that may be relevant for clinical decision making.

### 2.3. Patient Selection and Classification

The patients included in the study were those who underwent PGx testing to guide drug dosing prior to the initiation of therapy. The study population consisted of both hospitalized and outpatient individuals, depending on their clinical status and the need for inpatient care.

The PGx-guided drugs included in the study were as follows:

- Clopidogrel (for patients with ischemic stroke, myocardial infarction, or acute coronary syndrome).
- Fluoropyrimidines, tamoxifen, and irinotecan (for oncology patients starting chemotherapy).
- Azathioprine (for patients with autoimmune diseases requiring immunosuppressive therapy).
- Siponimod (for patients with secondary progressive multiple sclerosis—SPMS).

All patients, regardless of their hospitalization status, received the same PGx-guided treatments. Classification into hospitalized or outpatient groups was based solely on the clinical need for inpatient care, as follows:

1. Hospitalized patients required admission due to their underlying condition and the need for continuous monitoring, high-risk drug administration, or the management of severe immunosuppression or complications.
2. Outpatients were clinically stable individuals who did not require hospitalization but needed PGx testing before treatment initiation. These included, for example, patients with stable cardiovascular conditions, oncology patients initiating chemotherapy as outpatients, or individuals with autoimmune diseases or neurological disorders who could be managed without inpatient care.

### 2.4. Organization and Planning

Since 2021, various PGx tests have been progressively incorporated into daily clinical practice at our hospital. For each drug proposed for inclusion as a PGx-guided treatment, the PGx Unit, in collaboration with the corresponding medical departments likely to prescribe the drug, determines which genetic variants should be genotyped and what dosing recommendations should be issued.

The selection of genetic variants for routine clinical use is based on data collected from PharmGKB and publications from the Ubiquitous Pharmacogenomics Consortium (U-PGx) [28]. Only drug-response-related variants that meet the following criteria are considered: evidence level 1A/1B, an MAF greater than 0.1% or major clinical relevance, and the existence of established PGx dosing guidelines. Both CPIC and DPWG guidelines are reviewed, and therapeutic recommendations are issued accordingly. In cases of discrepancy between CPIC and DPWG guidelines, the PGx Unit harmonizes the information based on the best available scientific evidence after discussion between pharmacists and prescribing physicians, considering evidence strength and clinical applicability, with a preference for the most conservative recommendation to ensure patient safety. Table 1 summarizes the genetic variants, associated drugs, and phenotypes for which a dosing recommendation has been issued and subsequently implemented as a PGx test in our hospital.

#### Structure and Functioning of the PGx Unit

The PGx Unit is composed of a multidisciplinary team responsible for the implementation, execution, and interpretation of PGx testing in clinical practice. Established in 2021, the unit is integrated within the Hospital Pharmacy Service and is led by a hospital pharmacist. Its structure and roles are as follows:

- A nurse, responsible for collecting saliva samples from patients. Samples are collected at a PGx consultation within the Pharmacy Department for outpatients and at the hospital bedside for hospitalized patients. The nurse also ensures the proper identification and traceability of samples.
- A transport service, responsible for transferring samples from the hospital to an external laboratory.
- A laboratory team, which processes samples and generates genotyping results within 24 h, sending them to the hospital pharmacists.
- One pharmacist from the Pharmacy Department, responsible for interpreting genotypes, translating them into phenotypes, and providing personalized therapeutic recommendations to the medical departments requesting genetic testing. These recommendations are based on clinical practice guidelines, and, when necessary, discrepancies between different guidelines are harmonized using the available scientific evidence.

The medical departments involved in PGx testing include Neurology, Cardiology, Cardiovascular Intensive Care, Medical Oncology, Radiation Oncology, and Internal Medicine.

### 2.5. Sample and Data Collection

**Figure 1:**
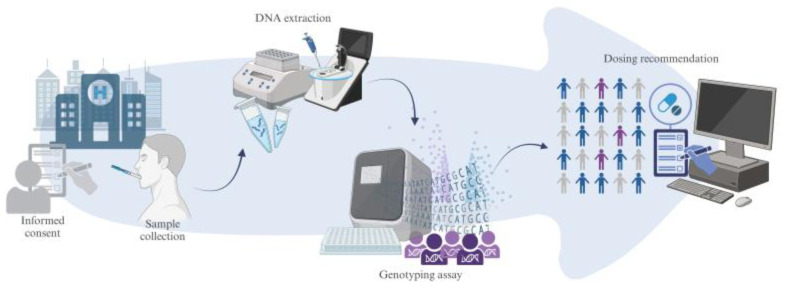
Clinical pharmacogenetics workflow.

When a drug with an available PGx test in our hospital is prescribed, physicians may either contact the PGx Unit directly or submit an electronic request for PGx testing. Upon receiving the request or call, the PGx Unit obtains the patient’s signed informed consent for genetic testing. Subsequently, a nurse collects a saliva sample using 2 to 4 sterile cotton swabs. DNA is extracted and stored in the laboratory for potential future analyses (Figure 1).

Sample collection is carried out at the bedside for hospitalized patients. For outpatients, an appointment at the PGx consultation unit is scheduled within 24 h of drug prescription.

Each patient is assigned a unique identification number (ID), which is used to label the collected cotton swabs. Personal data and ID matching are recorded in a secure, encrypted database accessible only to the pharmacists within the PGx Unit who are responsible for interpreting and reporting the results. Once labeled and documented, saliva samples are sent to the external laboratory for genotyping.

In addition to the biological sample, relevant data are systematically recorded, including the tested drug–gene interaction, the specific genetic variant (major nucleotide change and reference SNP ID), the hospital department requesting the PGx test, the date of saliva sample collection, and the patient’s resulting genotype and predicted phenotype.

To compare the allelic and genotypic frequencies of the tested variants in our population with those of a reference population, we retrieved data from the Iberian population included in the 1000 Genomes Project [26].

### 2.6. Genotyping and Results Reporting

For genotyping, DNA was isolated from saliva using standard procedures. The extraction followed a non-organic protocol based on proteinase K digestion and salting out, as described by Freeman et al. [29], with modifications introduced by Gómez-Martín A. et al. [30].

SNPs were genotyped using either predesigned TaqMan^®^ Genotyping Assays (Applied Biosystems, Foster City, CA, USA) or KASP genotyping assays (LGC Genomics, Hoddesdon, Hertfordshire, UK). TaqMan assays were performed on the QuantStudio™ 12K Flex Real-Time PCR System, while KASP assays were run on the Veriti™ 384-Well Thermal Cycler, both from Applied Biosystems (Foster City, CA, USA).

Genotyping is completed within 24 h of the PGx test request. The PGx Unit is responsible for interpreting the raw genotyping data, translating them into predicted phenotypes, and generating personalized dosing recommendations. A complete PGx report is then uploaded to the patient’s electronic medical record, where it becomes immediately available to the prescribing physician.

### 2.7. Clinical Decision Support System

To support the implementation of PGx in clinical practice, we developed an in-house CDSS. This tool is available as a mobile application—GenHUSC, accessible through major app stores—and is designed to assist healthcare professionals in the interpretation of PGx results.

The CDSS is based on recommendations from the DPWG and the Spanish Society of Pharmacogenetics and Genomics (SEFF). It translates genotypic information into phenotypic classifications and provides personalized therapeutic recommendations derived from validated clinical guidelines. Additionally, the system generates standardized clinical reports to ensure that prescribers receive clear, concise, and actionable PGx guidance.

The CDSS has been fully integrated into the hospital’s clinical workflow, supporting real-time decision making and enhancing the utility of PGx testing in routine patient care.

### 2.8. Data Management and Statistical Analysis

The genotypes for all SNPs analyzed by the PGx Unit are recorded for each patient. The allelic and genotypic frequencies of the studied variants were calculated and compared with those reported for the Iberian population in the 1000 Genomes Project. Statistical analyses were performed using R Commander. Comparisons were made using the chi-square test, or Fisher’s exact test when the expected frequency in at least one group of the contingency table was less than five.

LD, HWE, and haplotype analyses were carried out using the online tool SNPstats [31]. A *p*-value of < 0.05 was considered to be statistically significant.

## 3. Results

Since the first PGx test was performed in March 2021 by the PGx Unit within the Pharmacy Department of our hospital, a total of 1533 PGx tests have been conducted in 1386 unique patients. Overall, 28 different genetic variants have been genotyped, resulting in 6079 individual genotype results (see Table 2).

The PGx test for clopidogrel (*CYP2C19*) was requested 126 times, primarily by the following three hospital departments: Cardiology/Coronary Intensive Care Unit (*n* = 89) and Neurology (*n* = 37).

The PGx test for azathioprine, involving the *TPMT* and *NUDT15* genes, was requested 150 times by the Internal Medicine Department.

In the oncology and/or radiotherapy departments, a total of 1225 PGx tests were requested. These included genotyping for capecitabine/5-fluorouracil (5-FU) through *DPYD* testing (*n* = 1052), irinotecan through *UGT1A1* testing (*n* = 150), and tamoxifen through *CYP2D6* testing (*n* = 23).

Finally, the test for siponimod, which includes the genotyping of *CYP2C9*, was requested 32 times by the Neurology Department.

These PGx tests led to the following therapeutic recommendations: for clopidogrel/*CYP2C19*, 30 patients (23.81%) were classified as IM or PM; for azathioprine/*TPMT* and *NUDT15*, 15 patients (10.00%) carried variants associated with an increased risk of toxicity. Regarding capecitabine or 5-fluorouracil (5-FU)/*DPYD*, 47 patients were found to have a Gene Activity Score (GAS) lower than two. For irinotecan/*UGT1A1*, 23 patients (15.33%) were classified as PMs, and for tamoxifen/*CYP2D6*, 13 patients (56.52%) were identified as IMs.

Finally, in the case of siponimod/*CYP2C9*, four patients (12.5%) were classified as PMs and received a specific therapeutic recommendation. In total, 132 prescriptions (8.61%) would likely have resulted in suboptimal therapeutic outcomes without PGx guidance. Detailed distributions of genotypes, phenotypes, and related dosing recommendations are presented in Table 2.

### 3.1. Allelic and Genotypic Frequencies

The genotypic and allelic frequencies of the PGx variants selected for clinical implementation in our population were analyzed. Most of the variants did not show significant deviations from the HWE (*p* > 0.05), with the exception of *CYP2C19* rs12248560, which showed a slight deviation (*p* = 0.039), as shown in Table 3.

No patients in our cohort were found to carry the following variants: *CYP2C19* rs4986893 (*n* = 126); *UGT1A1* rs4148323 and rs35350960 (*n* = 150); and *CYP2D6* rs5030865 G > T (characterizing the *8 allele) and G > A (characterizing the *14 allele) and rs28371706 (*n* = 23).

Several PGx variants showed relevant MAFs in our cohort, indicating a high potential for clinical actionability. Specifically, the following SNPs had MAFs greater than 0.10: *CYP2C19* rs4244285 (MAF = 0.11) and rs12248560 (MAF = 0.18); *UGT1A1* rs3064744 (MAF = 0.36); *CYP2D6* rs3892097 (MAF = 0.24) and rs1065852 (MAF = 0.24); and *CYP2C9* rs1799853 (MAF = 0.17).

To further contextualize our findings, we compared the MAFs observed in our cohort with those reported by the 1000 Genomes Project for both the Iberian Peninsula and broader European (EUR) populations [26] . Notably, *CYP2C19* rs28399504 showed a significantly higher MAF in our cohort (MAF = 0.01) compared to the EUR population (MAF =0.001; *p* = 0.007). This difference may be explained by the fact that our cohort represents a regional subset of the broader reference population, potentially leading to genetic drift. This hypothesis is supported by the absence of significant differences when comparing our data with the Iberian Peninsula subgroup (*p* = 0.381).

### 3.2. Linkage Disequilibrium

LD was assessed using the following standard criteria: D’ > 0.5, r > 0.5, and *p* < 0.05. As observed in reference population datasets, the CYP2D*6 gene showed strong LD between the *4 (rs3892097) and *10 (rs1065852) star alleles, with values of D’ = 0.9996, r = 0.9433, and *p* = 0 (Appendix A). Similarly, *TPMT* alleles exhibited almost complete LD between TPMT*3B (rs1800460) and *3C (rs1142345), with D’ = 0.998, r = 0.9539, and *p* = 0.

In contrast, no significant LD was detected for variants within *CYP2C19*, *DPYD*, *UGT1A1*, or *CYP2C9*.

### 3.3. Analysis of Haplotype Frequencies

The haplotype frequencies observed for each gene tested by the PGx unit are presented in Appendix B. As previously shown in Table 2, a total of 132 patients (8.61%) carried genotypes associated with therapeutic recommendations according to established PGx guidelines.

For *CYP2D6*, the wildtype (wt) haplotype was present in 60.87% of patients, while haplotypes combining different *CYP2D6* variants were found in 39.13% of patients. The most prevalent among these was the combination of the wt, *4 (rs3892097), and *10 (rs1065852) alleles, which accounted for 15.22% of patients.

In the case of *CYP2C19*, 68.82% of patients carried the wt haplotype. The combination of the wt and *17 (rs12248560) allele was the most frequent variant haplotype, observed in 18.65% of the genotyped population.

For *CYP2C9*, 79.07% of patients carried the wt haplotype, with the most prevalent variant haplotype being the combination of the wt and *2 (rs1799853) alleles, which appeared in 13.12% of patients.

Finally, for both *TPMT* and *DPYD*, the wildtype genotype predominated, with frequencies of 95% and 97.62%, respectively. Haplotypes carrying variant alleles in these genes represented only a minor proportion of the studied population.

## 4. Discussion

In this study, we characterized several clinically relevant PGx drug–gene interactions under real-world clinical conditions. Our objective was to assess the clinical utility of PGx implementation by analyzing genotypes, phenotypes, and the resulting therapeutic recommendations. Additionally, we performed a comparative analysis of allelic and genotypic frequencies with reference populations, along with HWE, LD, and haplotype analyses.

It is important to note that real-world PGx implementation studies, such as the work by Stewart S. et al. [32], typically do not include formal sample size calculations. These studies focus on describing the adoption, feasibility, genotypic distributions, and clinical applicability of PGx testing rather than testing hypotheses or measuring outcomes. Our study followed a similar approach, aiming to document the integration of PGx into daily hospital practice from a descriptive and implementation science perspective.

Although there is no universal consensus regarding which genetic variants should be prioritized for PGx testing, the Association for Molecular Pathology (AMP) has published multiple gene-specific recommendations to help guide variant selection [33,34,35]. Our study aimed to integrate this guidance with real-world clinical data, identifying key PGx variants relevant to our local population among those most frequently cited in the literature. This strategy helps to mitigate potential biases associated with data generated in controlled research settings or from populations with different genetic backgrounds.

Our findings are consistent with the allele frequencies reported in other European populations, as documented in public pharmacogenomic resources such as 1000 Genomes [26] and GnomAD [27], supporting the generalizability of our implementation framework.

Our findings also reflect an implementation gap: many relevant drug–gene interactions are either not being tested in our hospital or are being outsourced to external laboratories. This is primarily because our institution has not yet incorporated several gene–drug interactions recently included in the Common Portfolio of Services of the Spanish National Health System, such as those related to statins, omeprazole, allopurinol, voriconazole, and certain antiepileptics. The PGx Unit at our hospital was established prior to the publication of this regulatory framework and initially prioritized those interactions with the greatest and most immediate clinical impact, particularly in response to physician requests.

Our long-term objective is to progressively expand the range of PGx tests available, aligning with national guidelines and clinical demand, with the ultimate goal of offering comprehensive PGx-guided prescribing across all relevant therapeutic areas.

### 4.1. Relevance of Implemented Drug–Gene Interactions

Regarding the clopidogrel/*CYP2C19* interaction, 30 patients (23.8%) were classified as IM or PM, and a change in antiplatelet therapy was recommended according to DPWG and CPIC dosing guidelines. Among these, three patients were categorized as IM/PM due to carrying the CYP2C19*4 allele (rs28399504), while no patients carried the CYP2C19*3 allele (rs4986893). Haplotype analysis revealed that nearly 100% of patients carried combinations of *CYP2C19* wt, *2 (rs4244285), and *17 (rs12248560). Notably, even the CYP2C19 *17/*17 genotype, associated with an UM phenotype, does not lead to a therapeutic recommendation in the current guidelines, despite being linked to an increased bleeding risk. Therefore, for clinical implementation of the *CYP2C19*/clopidogrel test, genotyping CYP2C19*2 (rs4244285) appears essential, while *3 (rs4986893) and *4 (rs28399504) may be useful. In contrast, routine testing for CYP2C19*17 is not necessary for clopidogrel therapy. Nonetheless, we acknowledge its clinical relevance for other drugs, such as voriconazole. In our hospital, *CYP2C19* testing is currently performed exclusively for clopidogrel prescription. To optimize resources, we store DNA samples and can rapidly (within 24 h) provide additional genotyping (e.g., CYP2C19*17) when needed and reassign the patient’s phenotype accordingly.

Clopidogrel was selected as one of the first drugs for PGx implementation due to its high clinical relevance in our setting. In fact, we have encountered patients who experienced two consecutive myocardial infarctions despite clopidogrel therapy, later attributed to reduced bioactivation confirmed by *CYP2C19* genotyping. These cases highlight the urgent clinical need to incorporate PGx-guided antiplatelet strategies. In contrast, for other CYP2C19 substrates such as voriconazole, genotyping is not yet routinely requested by clinicians nor implemented in our hospital. As our PGx program was launched in 2021, resources—scientific, technical, and economic—are limited. Therefore, we initially focused on gene–drug pairs with immediate therapeutic implications. Nevertheless, our workflow includes systematic DNA sample storage, enabling future testing expansion. As institutional resources grow and personalized medicine evolves, we expect to progressively increase the number of PGx variants integrated into routine clinical care.

For *TPMT*, 15 patients (10%) carried genotypes associated with therapeutic recommendations. LD analysis showed that TPMT*3B (rs1800460) and *3C (rs1142345) were almost completely linked: 11 patients carried both variants, none carried *3B alone, and 1 carried *3C only. According to current evidence, individuals with both SNPs are classified as TPMT *1/*3A [36]. Therefore, the genotyping of TPMT*3B may only be necessary to confirm the *3A allele when *3C is present [37].

No patients in our oncology cohort carried the DPYD*2A (rs3918290), UGT1A1*6 (rs4148323), or *27 (rs35350960) variants. However, considering the severe toxicities associated with these variants and the related healthcare costs, their inclusion in genotyping panels remains justified. In our institution, we genotype only *UGT1A1* rs3064744, which identifies TA repeat polymorphisms but does not distinguish between UGT1A1*28 and *37. These alleles contain seven and eight TA repeats, respectively (compared to six in UGT1A1*1), resulting in reduced transcription, expression, and enzymatic activity. Nevertheless, both CPIC and DPWG guidelines provide identical dosing recommendations for carriers of either *28 or *37 alleles.

Regarding *CYP2D6*/tamoxifen, no patients carried the CYP2D6*8 (rs5030865), *14 (rs5030865), or *17 (rs28371706) alleles. However, the gene is highly polymorphic, and most patients carried genotypes associated with variable enzyme activity and, consequently, variable tamoxifen response. Our results highlight CYP2D6*4 (rs3892097), *10 (rs1065852), and *41 (rs28371725) as particularly relevant for tamoxifen therapy. Clinical guidelines differ regarding the utility of *CYP2D6* genotyping for tamoxifen. While CPIC [24] and DPWG [20] support genotype-based dose adjustments, both the European Society for Medical Oncology (ESMO) [38] and the American Society of Clinical Oncology (ASCO) [39] advise against routine genotyping, citing inconsistent evidence. These discrepancies reflect the complexity and controversy surrounding *CYP2D6′s* role in tamoxifen efficacy. Until further conclusive evidence emerges, clinicians should consider available data carefully when making therapeutic decisions. Despite this lack of consensus, our institution decided to implement *CYP2D6* genotyping for tamoxifen based on the several following local considerations: (1) the clinical relevance of CYP2D6 in tamoxifen activation; (2) the existence of actionable recommendations from CPIC and DPWG; and (3) the integration of PGx into personalized treatment strategies in our oncology units, especially for patients with complex clinical profiles.

With respect to siponimod, the genotyping of CYP2C9*2 and *3 alleles is mandatory according to both the FDA and EMA. Siponimod is contraindicated in CYP2C9*3/*3 patients, and a reduced maintenance dose (1 mg/day) is recommended for those with *1/*3 or *2/*3 genotypes. However, CYP2C9*2 (rs1799853) genotyping alone does not translate into a specific recommendation unless analyzed alongside 3 (rs1057910). Notably, patients with *1/*2 or *2/*2 genotypes—classified as IM and PM phenotypes, respectively—do not receive distinct dosing guidance in current drug labels or guidelines. Although the CYP2C9*2 allele may hold PGx relevance, its independent clinical utility for siponimod dosing remains unclear [40].

### 4.2. Limitations

The main limitation of this study is that we did not include a comprehensive analysis of all drug–gene interactions with level 1A/1B evidence. Furthermore, not all reported SNPs within the included genes were genotyped. However, this is a common limitation in real-world clinical settings, where many Tier 1 variants defined by the AMP are not systematically tested across healthcare systems due to variability in local implementation strategies, clinical priorities, and resource constraints.

Another limitation is the lack of analysis of the association between PGx testing and clinical outcomes such as drug efficacy or toxicity. Nevertheless, the clinical relevance of the drug–gene interactions included in this study has been previously established and investigated in routine clinical practice.

Additionally, although we acknowledge the potential clinical impact of rare variants (MAF <1%), our study design precludes us from estimating their contribution to risk reduction or adverse event prevention.

As described in the Materials and Methods section (see Section 2.5, “Sample and Data Collection”), testing in our hospital is initiated only when requested by a physician prescribing a drug for which a PGx test is available in our unit. This reactive approach implies that, unless a test is actively requested, it is not performed—even if the test is available. Consequently, several clinically relevant drug–gene interactions, such as HLA-B*57:01/abacavir or *IFNL3*/ribavirin, are currently not being tested in our setting.

In addition, no formal sample size estimation was performed, as the primary aim of the study was descriptive. While this approach is consistent with other PGx implementation studies, it should be considered when interpreting the results—particularly in small subgroups such as tamoxifen and siponimod.

Finally, we were unable to compare the MAFs of UGT1A1*28 (rs8175347), CYP2D6*xN (gene duplication/multiplication), and CYP2D6*5 (gene deletion) with the 1000 Genomes Project dataset, as these data are not included in the database. However, the MAFs for CYP2D6*xN and *5 observed in our cohort were consistent with those reported for Caucasian populations by Ingelman-Sundberg et al. [41]. Similarly, our observed frequency for UGT1A1*28 was comparable to that reported for non-Finnish European populations in the GnomAD dataset [27,42].

### 4.3. Clinical Implementation of Pharmacogenetics

Currently, numerous drug–gene interactions are supported by the highest levels of evidence, are highlighted in regulatory agency communications (EMA/FDA), and have corresponding PGx-based dosing guidelines available.

Large-scale implementation projects have demonstrated the feasibility of integrating standardized PGx decision support tools into clinical practice [43]. Notably, genotype-guided treatments using PGx panels—such as 12-gene panels—have been shown to significantly reduce the incidence of clinically relevant toxicities [44]. However, there is still a clear need for strategies that facilitate the broader clinical adoption of PGx by healthcare institutions and providers, including improved compliance with regulatory and legal frameworks [43].

In this regard, applying a formal scoring system or decision matrix to prioritize gene–drug pairs might be useful for improving transparency and reproducibility.

In Spain, the publication of a brochure outlining the PGx tests included in the Common Portfolio of Services of the National Health System represents a major step forward. This document identifies the drug–gene interactions that should be offered for testing in public hospitals [45]. In parallel, the SEFF is currently developing national therapeutic guidelines based on this list, incorporating PGx information from official drug labels. These guidelines aim to standardize the clinical use of PGx by providing a curated list of relevant gene variants, phenotype classifications, and specific therapeutic recommendations based on genotype.

In our own clinical experience, we observed a high acceptance of PGx recommendations by physicians, particularly for *DPYD* testing. This did not negatively affect the efficacy of fluoropyrimidine-based chemotherapy [46]. Similarly, dose reductions of azathioprine in TPMT*1/*2, *1/*3A, or *1/*3C carriers were associated with a lower incidence of toxicity compared to TPMT*1/*1 individuals and with fewer subsequent dose adjustments—without compromising therapeutic efficacy.

However, challenges remain. Some healthcare professionals do not yet consider PGx as part of their clinical routine, and there are still gaps in education and awareness. Additionally, many PGx tests continue to be outsourced or paid for directly by the patient. Our data also support the hypothesis that additional genetic variants—beyond those currently tested—may influence azathioprine-related toxicity.

Importantly, rare PGx variants (MAF < 1%) play a critical role in clinical implementation. When designing a PGx panel for clinical use, rare variants may be excluded due to their low frequency. However, even extremely rare variants can lead to life-threatening adverse drug reactions, and their combined effect may impact a substantial proportion of patients. Therefore, while maintaining cost-effectiveness is essential, it is equally important to establish criteria for deciding which rare variants to include in clinical PGx panels to ensure both patient safety and therapeutic benefit.

## 5. Conclusions

Our findings support the growing need for studies that evaluate the clinical impact (in terms of efficacy improvement and/or toxicity reduction) and cost-effectiveness of implementing PGx in routine clinical practice. Future research should aim to harmonize implementation strategies—comparing single-drug testing approaches with broader multi-gene panel strategies—while identifying the most clinically relevant drug–gene interactions and genetic variants for testing. Additionally, laboratory protocols and clinical decision support systems must be validated to ensure the consistent and efficient application of PGx in the healthcare setting. Notable examples of coordinated efforts in this area include the U-PGx in Europe [27,33,43,44] and the MedeA initiative in Spain [47].

At present, there is no universally accepted criterion for determining which genetic variants should be prioritized for clinical PGx testing. However, resources such as PharmGKB provide curated classifications of PGx variants and are valuable references for guiding implementation. Additionally, the AMP has proposed standardization frameworks and gene-specific variant recommendations that support the global harmonization of clinical PGx testing criteria, and should also be considered when defining implementation strategies. Further research is needed to define clear clinical guidelines on which variants to test, in which patients, and under what circumstances.

In our experience, most PGx tests were requested by the oncology department, reflecting a greater awareness and clinical application of PGx in cancer treatment. Regarding the specific variants implemented at our center, we found that for the CYP2C19*17 allele, although associated with an UM phenotype, we do not recommend routine testing prior to clopidogrel prescription, as current guidelines do not provide therapeutic recommendations based on this genotype. Nonetheless, CYP2C19*17 may be relevant for other drugs, such as voriconazole, where increased enzyme activity can reduce drug exposure and compromise efficacy.

In the case of azathioprine, the genotyping of TPMT*3B may be considered only when necessary to confirm the presence of the TPMT*3A allele, given the strong linkage between TPMT*3B (rs1800460) and TPMT*3C (rs1142345). This approach can help to optimize resource use while maintaining clinical accuracy in phenotype prediction and dose adjustment.

With regard to siponimod, although current EMA and FDA guidelines recommend the genotyping of both CYP2C9*2 and CYP2C9*3 alleles, only the CYP2C9 *3/*3, *1/*3, and *2/*3 genotypes lead to dosing recommendations or contraindications. There is no specific guidance for CYP2C9*1/*2 or *2/*2 genotypes. Therefore, while CYP2C9*2 may have PGx relevance, its clinical utility in the context of siponimod prescribing remains limited. An alternative strategy could be to exclude CYP2C9*2 from routine testing for siponimod, or to update existing guidelines to address dosing in carriers of these genotypes.

Our implementation strategy was designed pragmatically, prioritizing gene–drug interactions with immediate therapeutic consequences and demonstrated clinical relevance. In resource-limited settings, focusing on variants that directly impact treatment decisions allows for the efficient and scalable integration of PGx into clinical workflows. The storage of DNA samples ensures future flexibility, enabling expanded testing as institutional resources and the scope of personalized medicine continue to grow.

While our study offers valuable insights into the clinical implementation of PGx, it is important to acknowledge certain limitations. No formal sample size estimation was conducted, as the study was descriptive and exploratory in nature. However, this should be considered when interpreting the data, especially in subgroups with a limited number of patients, such as those tested for tamoxifen and siponimod.

## Figures and Tables

**Table 1:** Available drug–gene interactions tested in our hospital and phenotypes with dosing recommendation.

Drug	Gene	Star Allele	Major Nucleotide Variation	dbSNP RS ID	Phenotypes
Clopidogrel	*CYP2C19*	*2	681G > A	rs4244285	PM/IM/NM/RM/UM
		*3	636G > A	rs4986893	
		*4A/B	1A > G	rs28399504	
		*17	−806C > T3	rs12248560	
Azathioprine	*TPMT*	*2	238G > C	rs1800462	PM/IM/NM
		*3B	460G > A	rs1800460	
		*3C	719A > G	rs1142345	
	*NUDT15*	*3	7973C > T	rs116855232	
Capecitabine/5-FU	*DPYD*	*2A	IVS14 + 1G > A (1905 + 1G > A)	rs3918290	GAS: 0;0.5;1;1.5;2
		*13	1679T > G	rs55886062	
		*-*	2846A > T	rs67376798	
		*-*	1236G > A	rs56038477	
Irinotecan	*UGT1A1*	*6	211G > A	rs4148323	PM/NM
		*27	686C > A	rs35350960	
		*28/*37	TA6 > TA7 or TA8	rs3064744	
Tamoxifen	*CYP2D6*	*xN	Gene multiplication	-	PM/IM/NM/UM
		*3	2549delA	rs35742686	
		*4	1846G > A	rs3892097	
		*5	Gene deletion	-	
		*6	1707delT	rs5030655	
		*8	1758G > T	rs5030865	
		*9	2615delAAG	rs5030656	
		*10	100C > T	rs1065852	
		*14A/B	1758G > A	rs5030865	
		*17	1023C > T	rs28371706	
		*41	2988G > A	rs28371725	
Siponimod	*CYP2C9*	*2	3608C > T	rs1799853	PM/IM/NM
		*3	42614A > C	rs1057910	

dbSNP RS ID: reference single-nucleotide polymorphism (SNP) identification from SNP database; PM: poor metabolizer; IM: intermediate metabolizer; NM: normal metabolizer; UM: ultrarapid metabolizer; 5-FU: 5 fluorouracil; GAS: Gene Activity Score.

**Table 2:** Detailed genotype–phenotype distribution and therapeutic recommendation assignment.

Phenotypes *n* (%)	Genotypes GENE* Carried Star Allele = Number of Patients	Therapeutic Recommendation
**Clopidogrel:** *CYP2C19* (*n =* 126)		
UM: 8 (6.35)	*CYP2C19* *17/*17 = 8	NO 76.19%
NM: 64 (50.79)	CYP2C19*1/*1 = 64	
RM: 24 (19.05)	CYP2C19*1/*17 = 24	
IM: 28 (22.22)	CYP2C19*1/*2 = 18; *1/*4 = 3; *2/*17 = 7	YES 23.81%
PM: 2 (1.59)	CYP2C19*2/*2 = 1; *2/*4 = 1	
**Azathioprine:** *TPMT* and *NUDT15* (*n =* 150)		
NM: 135 (90.00)	TPMT*1/*1 = 135; NUDT15*1/*1 = 135	NO 90.00%
IM: 15 (10.00)	TPMT*3B/*3C (TPMT*1/*3A) =11; *1/*2 = 1; *1/*3C = 1. NUDT15*1/*3 = 2	YES 10.00%
PM: 0 (0)	*-*	
**Capecitabine/5-FU**:*DPYD* (*n* = 1052)		
GAS 2: 1005 (95.53)	DPYD*1/*1 = 1005	NO 95.53%
GAS 1.5: 46 (4.37)	DPYD*1/rs56038477 = 37; *1/rs67376798 = 9	YES 4.47%
GAS 1: 1 (0.01)	DPYD*1/*13 = 1	
**Irinotecan:***UGT1A1* (*n* = 150)		
NM: 65 (43.33)	UGT1A1*1/*1 = 65	NO 84.67%
IM: 62 (41.33)	UGT1A1*1/*28 = 62	
PM: 23 (15.33)	UGT1A1*28/*28 = 23	YES 15.33%
**Tamoxifen:***CYP2D6* (*n* = 23)		
NM: 9 (39.13)	CYP2D6*1/*1= 6; *1/*41 = 2; *1/*10 = 1;	NO 43.48%
UM: 1 (4.35)	CYP2D6*1/*xN = 1	
IM: 13 (56.52)	CYP2D6*1/*5 = 1; *1/*3 = 1; *4/*10 = 6; *4/*xN/*10 = 1; *4/*10/*6 = 1; *4/*10/*9 = 2; *4/*10/*41 = 1	YES 56.52%
PM: 0 (0)	*-*	
**Siponimod:***CYP2C9* (*n* = 32)		
NM: 20 (62.5)	CYP2C9*1/*1 = 20	NO 87.50%
IM: 8 (25.0)	CYP2C9*1/*2 = 8	
PM: 4 (12.5)	CYP2C9*2/*3 = 4	YES 12.50%
	**TOTAL:** *n* = 1533	
*n* = 1401 (91.39)	*-*	NO 91.39%
*n* = 132 (8.61)	*-*	YES 8.61%

PM: poor metabolizer; IM: intermediate metabolizer; NM: normal metabolizer; UM: ultrarapid metabolizer; GAS: Gene Activity Score.

**Table 3:** Genotypic and allelic frequencies in our population, Hardy–Weinberg equilibrium test, and MAF comparison with 1000 Genomes Project.

Requested PGx Test *n* = 1533	*SNP* *(star allele)*	Major Nucleotide Variation	dbSNP RS ID	Our Population *n =* 1567 ⴕ					IBS *n =* 107	EUR *n =* 503
				Wt *n* (%)	Het *n* (%)	Hom *n* (%)	MAF	*p*-Value (H-W)	*p*-Value	*p*-Value
**Clopidogrel ** ***n =*** 126	CYP2C19*2	681G > A	rs4244285	99 (78.57)	26 (20.63)	1 (0.80)	0.111	1	0.275	0.185
	CYP2C19*3	636G > A	rs4986893	126 (100)	0 (0)	0 (0)	0	-	1 *	1 *
	CYP2C19*4A/B	1A > G	rs28399504	122 (96.82)	4 (3.18)	0 (0)	0.016	1	0.381	**0.007**
	CYP2C19*17	−806C > T3	rs12248560	87 (69.05)	31 (24.60)	8 (6.35)	0.187	0.039	0.444	0.200
**Azathioprine ** ***n =*** 150	TPMT*2	238G > C	rs1800462	149 (99.33)	1 (0.67)	0 (0)	0.003	1	0.573 *	1 *
	TPMT*3B	460G > A	rs1800460	139 (92.67)	11 (7.33)	0 (0)	0.037	1	0.755	0.430
	TPMT*3C	719A > G	rs1142345	138 (92.00)	12 (8.00)	0 (0)	0.04	1	0.908	0.330
	NUDT15*3	7973C > T	rs116855232	148 (98.67)	2 (1.33)	0 (0)	0.007	1	0.513 *	0.228 *
**Capecitabine/5-FU ** ***n =*** 1052	DPYD*2A	IVS14 + 1G > A	rs3918290	1049 (99.71)	3 (0.29)	0 (0)	0.001	1	1 *	0.122 *
	DPYD*13	1679T > G	rs55886062	1051 (99.90)	1 (0.10)	0 (0)	0.001	1	1 *	0.542 *
	*-*	2846A > T	rs67376798	1043 (99.14)	9 (0.86)	0 (0)	0.004	1	1 *	0.328
	*-*	1236G > A	rs56038477	1015 (96.48)	37 (3.52)	0 (0)	0.018	1	0.907	0.721
**Irinotecan ** ***n =*** 150	UGT1A1*6	211G > A	rs4148323	150 (100)	0 (0)	0 (0)	0	-	1 *	0.604 *
	UGT1A1*27	686C > A	rs35350960	150 (100)	0 (0)	0 (0)	0	-	1 *	1 *
	UGT1A1*28/*37	TA6 > TA7 or TA8	rs3064744	65 (43.34)	62 (41.33)	23 (15.33)	0.36	0.22	NA	0.147
**Tamoxifen ** ***n =*** 23	CYP2D6*xN	Multiplication	-	21 (87.5)	2 (12.5)	0 (0)	0.043	1	NA	NA
	CYP2D6*3	2549delA	rs35742686	22 (91.66)	1 (8.33)	0 (0)	0.022	1	0.449 *	0.594 *
	CYP2D6*4	1846G > A	rs3892097	12 (52.17)	11 (47.83)	0 (0)	0.239	0.28	0.115	0.366
	CYP2D6*5	Deletion	-	22 (91.66)	1 (8.33)	0 (0)	0.022	1	NA	NA
	CYP2D6*6	1707delT	rs5030655	22 (91.66)	1 (8.33)	0 (0)	0.022	1	0.323 *	0.613 *
	CYP2D6*8	1758G > T	rs5030865	23 (100)	0 (0)	0 (0)	0	-	1 *	1 *
	CYP2D6*9	2615delAAG	rs5030656	21 (87.5)	2 (12.5)	0 (0)	0.043	1	0.359 *	0.349 *
	CYP2D6*10	100C > T	rs1065852	11 (47.83)	12 (52.17)	0 (0)	0.239	0.27	0.293	0.538
	CYP2D6*14A/B	1758G > A	rs5030865	23 (100)	0 (0)	0 (0)	0	-	1 *	1 *
	CYP2D6*17	1023C > T	rs28371706	23 (100)	0 (0)	0 (0)	0	-	1 *	1 *
	CYP2D6*41	2988G > A	rs28371725	20 (86.95)	3 (13.05)	0 (0)	0.065	-	0.775 *	0.793 *
**Siponimod *****n =*** 32	CYP2C9*2	3608C > T	rs1799853	21 (65.62)	11 (34.38)	0 (0)	0.172	0.56	0.530	0.267
	CYP2C9*3	42614A > C	rs1057910	28 (87.5)	3 (9.38)	1 (3.12)	0.078	0.15	0.879	0.868

PGx: pharmacogenetic; SNP: single-nucleotide polymorphism; Wt: wildtype genotype; Het: heterozygous genotype; Hom: recessive homozygous genotype; MAF: minor allele frequency; H-W: Hardy–Weinberg equilibrium; IBS: population from the Iberian Peninsula included as subpopulation in the 1 000 Genomes Project. * Fisher exact test. ⴕ See in the first column the ”*n*” genotyped for each gene.

## Data Availability

The raw data supporting the conclusions of this article will be made available by the authors on request.

## Abbreviations

The following abbreviations are used in this manuscript:

5-FU	5-Fluorouracil
ADE	Adverse drug event
CPIC	Clinical Pharmacogenetics Implementation Consortium
CYP2C19	Cytochrome P-450 family 2 subfamily C member 19
CYP2D6	Cytochrome P450 family 2 subfamily D member 6
DNA	Deoxyribonucleic acid
DPYD	Dihydropyrimidine dehydrogenase
DPWG	Dutch Pharmacogenetics Working Group
EMA	European Medicines Agency
EUR	European
FDA	Federal Drug Administration
GOF	Gain of function
HET	Heterozygous genotype
HOM	Recessive homozygous genotype
H-W	Hardy–Weinberg
IBS	Iberian peninsula
ID	Identification number
IM	Intermediate metabolizer
LD	Linkage disequilibrium
LOF	Loss of function
MAF	Minor allele frequency
NM	Normal metabolizer
PGx	Pharmacogenetics
PM	Poor metabolizer
SNP	Single-nucleotide polymorphism
TPMT	Thiopurine methyltransferase
UGT1A1	Uridine diphosphate glucuronosyltransferase 1 family, polypeptide A1
UM	Ultrarapid metabolizer
WT	Wildtype

## Appendix A

**Table A1:** Linkage disequilibrium analysis for each gene.

***CYP2D6* *n* = 23**							
	***CYP2D6*3***	***CYP2D6*4***	***CYP2D6*5***	***CYP2D6*6***	***CYP2D6*9***	***CYP2D6*10***	***CYP2D6*41***
***CYP2D6*XN***	0.9286 −0.0295 0.8414	0.046 0.0175 0.9056	0.9286 −0.0295 0.8414	0.9286 −0.0295 0.8414	0.9774 −0.0444 0.7632	0.143 −0.0181 0.9023	0.9849 −0.0555 0.7068
***CYP2D6*3***		0.987 −0.0825 0.5759	0.8571 −0.019 0.8972	0.8571 −0.019 0.8972	0.9286 −0.0295 0.8414	0.9881 −0.0875 0.5528	0.9524 −0.0375 0.7992
***CYP2D6*4***			0.987 −0.0825 0.5759	0.9959 0.2648 0.0725	0.9987 0.3798 **0.01**	0.9996 0.9433 **0**	0.9971 0.1477 0.3166
***CYP2D6*5***				0.8571 −0.019 0.8972	0.9286 −0.0295 0.8414	0.9881 −0.0875 0.5528	0.9524 −0.0375 0.7992
***CYP2D6*6***					0.9286 −0.0295 0.8414	0.9958 0.2499 0.0901	0.9524 −0.0375 0.7992
***CYP2D6*9***						0.9987 0.3584 **0.0151**	0.9849 −0.0555 0.7068
***CYP2D6*10***	D´ r *p*-value						0.9965 −0.1564 0.2889
***DPYD* *n* = 1502 **							
	**1679T > G**	**2846A > T**	**1236G > A**				
**1905+1G > A**	0.1447 0.0835 **1 × 10^−4^**	0.0517 0.0298 0.1718	0.0388 0.011 0.615				
**1679T > G**		0.1422 0.0473 **0.03**	0.1306 0.0213 0.3288				
**2846A > T**			0.3307 −0.0029 0.8942				
***CYP2C19* *n* = 126 **							
	***CYP2C19*4***	***CYP2C19*17***					
***CYP2C19*2***	0.0351 0.0126 0.8412	0.9978 0.1689 **0.0073**					
***CYP2C19*4***		0.9731 −0.0592 0.3476					
***TPMT/NUDT15* *n* = 150 **							
	***TPMT*3B***	***TPMT*3C***	***NUDT15***				
***TPMT*2***	0.4195 −0.0047 0.9347	0.4678 −0.0055 0.9238	0.0147 0.0104 0.8572				
***TPMT*3B***		0.998 0.9539 **0**	0.8077 0.0129 0.8231				
***TPMT*3C***			0.8238 0.0138 0.8114				
***CYP2C9* *n* = 32 **							
	***CYP2C9*3***						
***CYP2C9*2***	0.421 0.269 **0.0314**						

D’, r, and *p*-values are shown for each SNP–SNP combination in *CYP2D6*, *TPMT/NUDT15*, CYP2C9 *CYP2C19,* and *DPYD* genes. Bold letters mean *p* < 0.05.

## Appendix B

**Table A2:** Haplotype frequencies estimations for each gene.

	*CYP2D6* (*n* = 23) *star allele and Major allele > minor allele									
	*XN	*3 A > del	*4 G > A	*5 (del)	*6 T > del	*9 AAG > del	*10 C > T	*41 G > A	Total	Cumulative Frequency
*1*	*G*	*A*	*G*	*A*	*T*	*AAG*	*C*	*G*	*0.6087*	*0.6087*
*2*	*G*	*A*	*A*	*A*	*T*	*AAG*	*T*	*G*	*0.1522*	*0.7609*
*3*	*G*	*A*	*G*	*A*	*T*	*AAG*	*C*	*A*	*0.058*	*0.8188*
*4*	*G*	*A*	*A*	*A*	*T*	*del*	*T*	*G*	*0.0435*	*0.8623*
*5*	*xN*	*A*	*G*	*A*	*T*	*AAG*	*C*	*G*	*0.029*	*0.8913*
*6*	*G*	*A*	*A*	*A*	*del*	*AAG*	*T*	*G*	*0.0217*	*0.913*
*7*	*G*	*A*	*G*	*A*	*T*	*AAG*	*T*	*G*	*0.0217*	*0.9348*
*8*	*G*	*A*	*G*	*del*	*T*	*AAG*	*C*	*G*	*0.0217*	*0.9565*
*9*	*G*	*del*	*G*	*A*	*T*	*AAG*	*C*	*G*	*0.0217*	*0.9783*
*10*	*xN*	*A*	*A*	*A*	*T*	*AAG*	*T*	*G*	*0.0145*	*0.9927*
*11*	*G*	*A*	*A*	*A*	*T*	*AAG*	*T*	*A*	*0.0073*	*1*
***DPYD* (*n* = 1502)** **Major nucleotide variation**										
					**1905 + 1G > A**	**1679** **T > G**	**2846** **A > T**	**1236** **G > A**	**Total**	**Cumulative** **frequency**
				*1*	*C*	*A*	*T*	*C*	*0.9762*	*0.9762*
				*2*	*C*	*A*	*T*	*T*	*0.0176*	*0.9938*
				*3*	*C*	*A*	*A*	*C*	*0.0043*	*0.9981*
				*4*	*T*	*A*	*T*	*C*	*0.0014*	*0.9995*
				*5*	*C*	*C*	*T*	*C*	*5 × 10^−4^*	*1*
***TPMT/NUDT15* (*n* = 150)** ***star allele and Major allele > minor allele**										
					***TPMT*2*** ***G > C***	***TPMT*3B*** ***G > A***	***TPMT*3C*** ***A > G***	***NUDT15*** ***C > T***	**Total**	**Cumulative** **frequency**
				1	G	G	A	C	0.95	0.95
				2	G	A	G	C	0.0367	0.9867
				3	G	G	G	T	0.0067	0.9933
				4	C	G	A	C	0.0033	0.9967
				5	G	G	G	C	0.0033	1
***CYP2C9* (*n* = 32)** ***star allele and Major allele > minor allele**										
							****2*** ***C > T***	****3*** ***A > C***	**Total**	**Cumulative** **frequency**
						***1***	***C***	*A*	*0.7907*	*0.7907*
						*2*	*T*	*A*	*0.1312*	*0.9219*
						*3*	*T*	*C*	*0.0407*	*0.9625*
						*4*	*C*	*C*	*0.0375*	*1*
***CYP2C19* (*n* = 126)** ***star allele and Major allele > minor allele**										
						****2*** ***G > A***	****4*** ***A > G***	****17*** ***C > T***	**Total**	**Cumulative** **frequency**
					***1***	*G*	*A*	*C*	*0.6882*	*0.6882*
					*2*	*G*	*A*	*T*	*0.1865*	*0.8747*
					*3*	*A*	*A*	*C*	*0.1094*	*0.9841*
					*4*	*G*	*G*	*C*	*0.0142*	*0.9983*
